# A Comprehensive Review of Crop Chlorophyll Mapping Using Remote Sensing Approaches: Achievements, Limitations, and Future Perspectives

**DOI:** 10.3390/s25082345

**Published:** 2025-04-08

**Authors:** Xuan Li, Bingxue Zhu, Sijia Li, Lushi Liu, Kaishan Song, Jiping Liu

**Affiliations:** 1 State Key Laboratory of Black Soils Conservation and Utilization, Northeast Institute of Geography and Agroecology, Chinese Academy of Sciences, Changchun 130102, China; lixuan@iga.ac.cn (X.L.); lisijia@iga.ac.cn (S.L.); liulushi@iga.ac.cn (L.L.); songkaishan@iga.ac.cn (K.S.); 2 College of Tourism and Geography, Jilin Normal University, Siping 136000, China; ljp@jlnu.edu.cn

**Keywords:** chlorophyll content, hyperspectral imaging, machine learning, radiative transfer models, precision agriculture

## Abstract

Chlorophyll absorbs light energy and converts it into chemical energy, making it a crucial biochemical parameter for monitoring vegetation health, detecting environmental stress, and predicting physiological states. Accurate and rapid estimation of canopy chlorophyll content is crucial for assessing vegetation dynamics, ecological changes, and growth patterns. Remote sensing technology has become an indispensable tool for monitoring vegetation chlorophyll content since 2015, with more than 50 research papers published annually, contributing to a substantial body of case studies. This review discusses remote sensing technologies currently used for estimating vegetation chlorophyll content, focusing on four key aspects: the acquisition of reference datasets, the identification of optimal spectral variables, the selection of estimation models, and the analysis of application scenarios. The results indicate that spectral bands in the visible and red-edge regions (e.g., 530 nm, 670 nm, and 705 nm) provide high prediction accuracy. Machine learning methods, such as random forest and support vector regression, exhibit excellent performance, with determination coefficients (R^2^) typically exceeding 0.9, although overfitting remains an issue. Although radiative transfer models are slightly less accurate (R^2^ = 0.6–0.8), they provide greater interpretability. Hybrid models integrating machine learning and radiative transfer show strong potential to balance accuracy and generalizability. Future research should improve model generalizability for different vegetation types and environmental conditions and integrate multi-source remote sensing data to improve spatial and temporal resolution. Combining physical models with data processing methods, such as artificial intelligence, can improve scalability, cost-effectiveness, and real-time monitoring capabilities.

## 1. Introduction

Chlorophyll is a critical biochemical parameter for crops [1,2] and a good indicator of plant nutritional stress, photosynthetic capacity, development, and senescence stages [3,4,5]. The chlorophyll content of crops directly affects nitrogen content and physiological state. It is crucial for dynamic monitoring of crop growth, pest and disease management, crop yield, and predicting crop maturity [6,7,8]. Since the 1970s, remote sensing technology has become essential for large-scale estimation of crop biochemical parameters [9,10] due to its broad coverage [11,12], rapid information acquisition, high efficiency, and convenience [13].

Chlorophyll content inversion is performed by determining the quantitative relationship between spectral features of vegetation and the chlorophyll content [5,14]. Current remote sensing data sources include ground-based spectral measurements and data acquired from drones and satellite imagery [15,16]. Ground-based spectrometers, e.g., instruments from Analytical Spectral Devices (ASDs) and Spectra Vista Corporation (SVC), provide detailed spectral data from crop components, including leaves, maize ears, and canopies [12,17,18,19], but their coverage is limited. Satellite remote sensing data provide large-scale, long-term data on vegetation growth, although the resolution is relatively low. Drones are an emerging remote sensing platform to capture high-resolution images and spectral data of crops [18,20], offering flexibility and quick response. The accuracy of crop chlorophyll content inversion can be improved by integrating multisource data with appropriate inversion algorithms and models [21,22]. These data sources have advantages and disadvantages, and they are complementary.

Chlorophyll exhibits two absorption peaks in the visible light spectrum (640–660 nm in the red region and 430–450 nm in the blue–violet region) [14,23]. In the near-infrared spectrum, the leaf structure and thickness affect leaf reflectance. The spectral reflectance increases rapidly in the red-edge region and is highly correlated with the leaf chlorophyll content; thus, this region is commonly used for estimating or inverting chlorophyll content in plant leaves [24]. Among the four methods for estimating crop chlorophyll content (Parametric regression methods, Nonparametric regression methods, Physically based methods, and Mixed methods) [25,26], constructing statistical relationships between vegetation reflectance, vegetation indices, and chlorophyll content is the most widely used [27]. Inversion of crop chlorophyll content using mathematical methods, such as iterative algorithms, lookup table algorithms, neural network algorithms, support vector machine (SVM), and random forest, is simple and accurate. However, it lacks clear physical meaning and has low spatiotemporal scalability and generalization ability, limiting its application value [28]. In contrast, physical models based on radiative transfer simulate the spectral characteristics of leaves by describing photon scattering and absorption within or between leaves [29,30]. They describe the relationship between spectral reflectance and chlorophyll content, improving the accuracy of chlorophyll content inversion [31]. Although the physical model has clear physical meaning, its inversion process relies on complex parameterization process, which may be limited by high computational complexity in heterogeneous surface or large-scale applications [32]. Due to technological advancements, these two types of models can be combined. Coupled models have improved the accuracy of chlorophyll content estimation [33].

Although the spectral inversion method of chlorophyll content is relatively mature, it still has problems of insufficient accuracy and weak model generalization ability in complex environments (such as heterogeneous vegetation canopies and multi-crop mixed areas) [34]. This review aimed to systematically analyze the limitations of existing methods and propose a path to improve the inversion accuracy through multi-source data fusion and hybrid model optimization.

**Figure 1:**
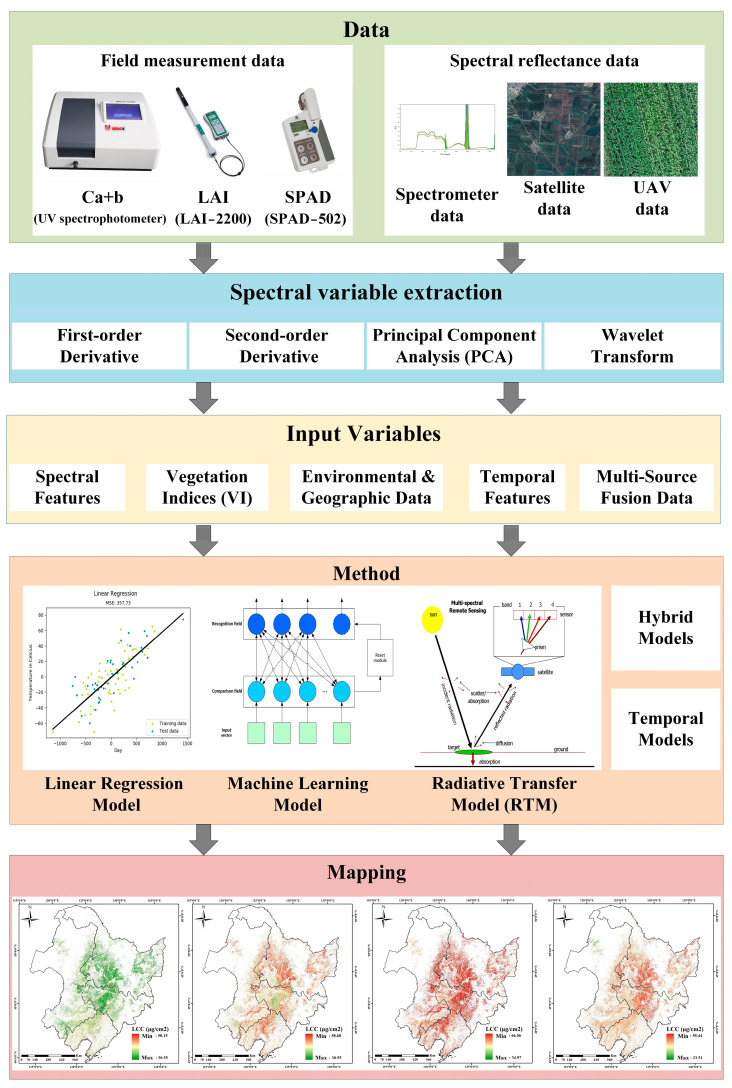
Flowchart of remote sensing methods for estimating crop chlorophyll content.

Estimating crop chlorophyll content using remote sensing data is a complex process. The scope and complexity of the study area influence the selection of sample plots, extraction of remote sensing data, and the modeling algorithms for chlorophyll content estimation. Figure 1 shows a flowchart for estimating crop chlorophyll content. We used these steps to guide this review. We provided a bibliometric analysis of research in this field (Section 2), an overview of extracting and selecting chlorophyll-related variables in crops (Section 3), a summary and analysis of algorithms used for chlorophyll content estimation (Section 4), a comprehensive evaluation of the performance of different remote sensing inversion models, optimization suggestions and future development directions (Section 5), as well as the conclusions and outlook (Section 6).

## 2. Results of Bibliometric Analysis

**Figure 2:**
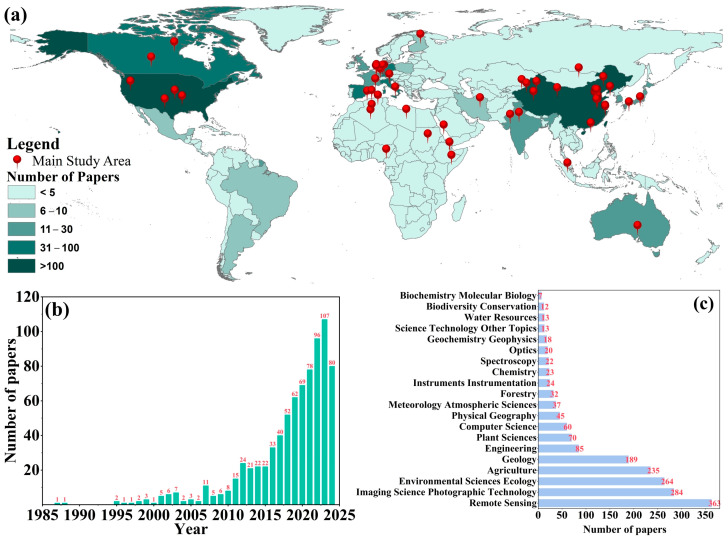
Bibliometric analysis results. (**a**) Study area and number of papers, (**b**) publications, (**c**) research areas.

A total of 788 relevant papers were retrieved from the Web of Science database using core keywords, such as chlorophyll content, remote sensing, crops, data sources, and inversion. Research on crop chlorophyll remote sensing inversion has primarily focused on temperate and subtropical regions (Figure 2a). The United States, China, Canada, Spain, and Germany are the main contributing countries, with the United States and China leading in the number of studies, indicating that these countries with strong agricultural production are highly focused on remote sensing technology. The number of papers has increased exponentially in recent years, especially after 2015, with annual publications stabilizing at over 50 papers (Figure 2b). This growth can be attributed to the rapid development of hyperspectral remote sensing, drone platforms, and machine learning technologies, which have improved research efficiency and precision. Research areas include remote sensing, environmental science and ecology, agriculture, imaging science, and photographic technology. The dominance of “remote sensing” (Figure 2c) suggests this technology is the core tool for chlorophyll inversion research. Additionally, its application value has increased in agricultural production and environmental protection. This trend shows that remote sensing technology has become an important tool in crop chlorophyll inversion. Future research should extend to developing countries and ecologically sensitive areas to examine potential applications in broader agricultural and environmental contexts.

## 3. Spectral and Vegetation Variables Related to Chlorophyll Estimation

The data sources for estimating crop chlorophyll content include ground spectral data, airborne remote sensing data, and satellite remote sensing data. The data selection should comprehensively consider the application scenario of chlorophyll estimation and the required accuracy level [35].

### 3.1. Remote Sensing Image Data Sources for Chlorophyll Content Estimation

**Figure 3:**
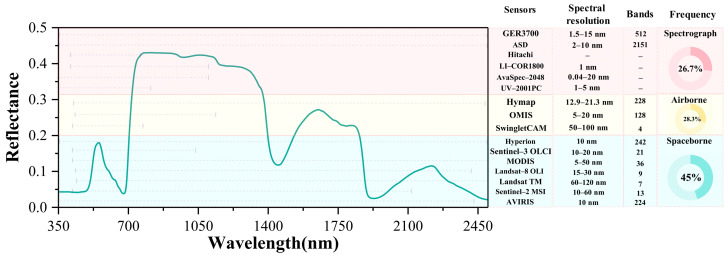
Spectral coverage and band utilization frequency of ground-based, airborne, and spaceborne sensors (frequency defined as the percentage of studies using each wavelength).

Remote sensing data for chlorophyll estimation can be categorized into ground-based, airborne, and spaceborne sources [35]. each differing in wavelength coverage and sensor characteristics (Figure 3). Ground spectrometers (e.g., GER3700, ASD, LI-COR1800) cover a broad spectral range (350–2500 nm), enabling fine red-edge detection and are commonly used for field validation, representing 26.7% of studies [36]. However, their limited coverage, low efficiency, and high cost hinder large-scale applications [37].

Airborne sensors (e.g., MicaSense RedEdge, HyMap, OMIS) provide high-resolution spectral and spatial data suitable for small- to medium-scale monitoring [38]. Hyperspectral systems like HyMap (450–2500 nm, 126 bands) and multispectral systems like RedEdge (5 bands, including red-edge and NIR) are widely used due to their flexibility and cost-effectiveness [39].

Spaceborne sensors, applied in 45% of studies, support regional- to global-scale monitoring due to wide coverage and revisit frequency. Sentinel-2 MSI (13 bands, 10–60 m), Landsat-8 OLI (11 bands, 30 m), MODIS (36 bands, 250–1000 m), and Hyperion (220 bands, 400–2500 nm) are commonly used [28,40,41]. Despite resolution constraints, their large-scale observation capability is vital for chlorophyll mapping.

With growing demand for chlorophyll inversion, multi-source data fusion has become essential. By integrating complementary data, such as Sentinel-2 with ground-based hyperspectral sensors [42], Landsat-8 with UAV images, or MODIS with in situ measurements [43], fusion approaches enhance both inversion accuracy and spatial–temporal robustness.

As the demand for crop chlorophyll remote sensing inversion has increased, multisource remote sensing data fusion has become essential to improving accuracy. This method integrates the strengths of different platforms, combining macro-scale coverage with local observations. Comprehensive technical support is provided for chlorophyll inversion [44]. High-resolution spatial data from Sentinel-2 have been combined with high-resolution spectral data from ground-based hyperspectral sensors for inversion to analyze the spatiotemporal dynamics of crop chlorophyll, significantly enhancing inversion accuracy and supporting crop health assessment. Time-series data from Landsat-8 have been integrated with high-resolution drone images to estimate the chlorophyll content of different crop types, improving the robustness and universality of inversion models [45].

Multi-source remote sensing data fusion has been shown to significantly improve the accuracy and robustness of chlorophyll content inversion. For example, the integration of UAV multispectral, thermal, and RGB data using a stacking ensemble learning approach increased the coefficient of determination (R^2^) from 0.699 to 0.754, while reducing the relative RMSE to 8.36–9.47% [46]. The combination of Landsat-8 imagery and GEDI LiDAR data, modeled with random forest, yielded the highest performance among the tested models, with an R^2^ of 0.94 and RMSE of 0.18 g/m^2^ [47]. Similarly, by fusing UAV hyperspectral data with ground measurements and simulating Sentinel-2A spectral reflectance, the XGBoost MIC model achieved a test R^2^ of 0.837 and RMSE of 3.250 mg/m^2^, representing a substantial improvement over using UAV data alone (R^2^ = 0.582) [48]. These findings confirm the advantages of combining complementary data sources to enhance chlorophyll estimation across different platforms and spatial scales.

### 3.2. Spectral Variables Associated with Chlorophyll Content

**Figure 4:**
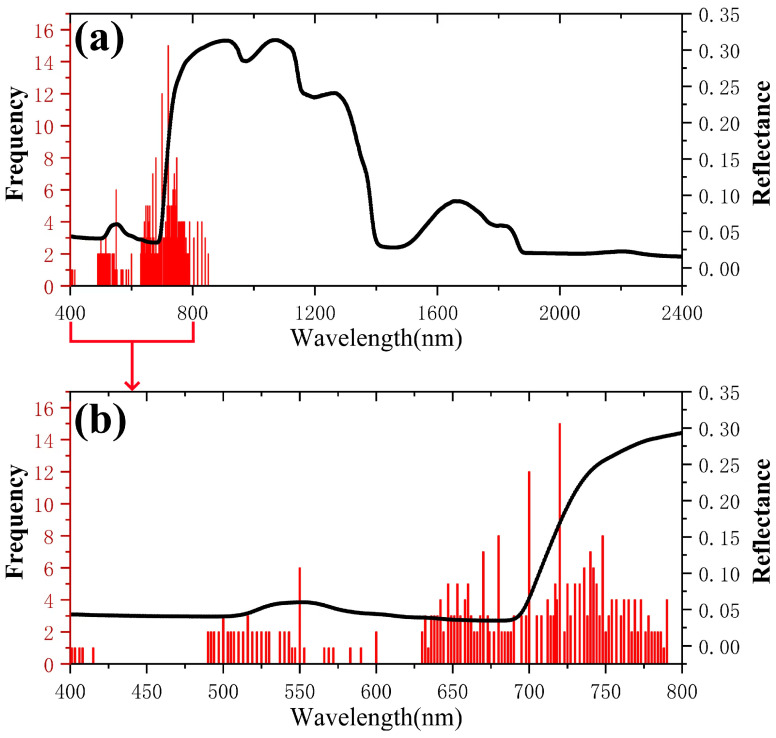
Frequency of using wavelengths for hyperspectral inversion of chlorophyll content. (**a**) Wavelength frequency of 200–2400 nm, (**b**) local amplification at 400–800 nm.

The frequency of using different wavelengths for crop chlorophyll inversion using hyperspectral data is shown in Figure 4. Spectral bands suitable for estimating crop chlorophyll content range from 470 nm to 790 nm [49]. Bands with a frequency of use greater than or equal to 5 are located in the 400–800 nm range, particularly in the 450–750 nm range. The blue (450–500 nm) and red (around 680 nm) bands are the primary chlorophyll absorption bands, reflecting the chlorophyll content in the leaves, and the red-edge band (670–750 nm) is commonly used due to its sensitivity to the chlorophyll content [24]. The green band (550 nm) has lower chlorophyll absorption, but the “green peak” has been used to reflect dynamic changes in chlorophyll content [14]. Healthy plants have high reflectance in the near-infrared region (700–1100 nm). Thus, a higher chlorophyll content is correlated with higher near-infrared reflectance.

### 3.3. Vegetation Indices

**Figure 5:**
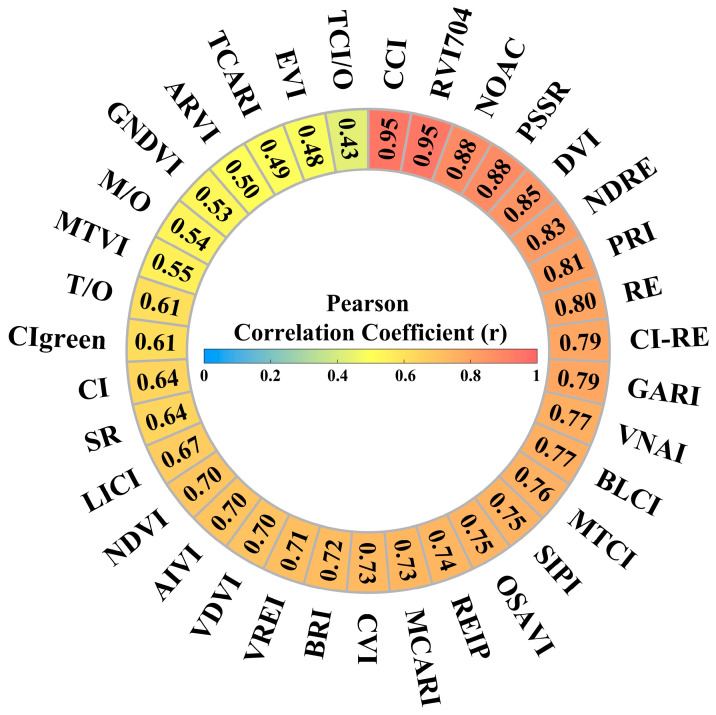
Correlations between VIs and chlorophyll content. Note: M/O = MCARI/OSAVI, TCI/O = TCI/OSAVI, T/O = TCARI/OSAVI.

Vegetation indices reflect the growth status of vegetation. They are straightforward to implement, have a low computational load for processing, and have been widely used in estimating crop chlorophyll content [50]. Common vegetation indices include the Normalized Difference Vegetation Index (NDVI), Perpendicular Vegetation Index (PVI), Difference Vegetation Index (DVI), Ratio Vegetation Index (RVI), and Soil-Adjusted Vegetation Index (SAVI) [51]. These vegetation indices are correlated with plant parameters, such as leaf chlorophyll content and leaf area index (LAI). NDVI tends to saturate when the leaf area index (LAI) is high, resulting in decreased sensitivity to high chlorophyll content. To this end, studies have proposed improved indices (such as EVI and CIred-edge) to alleviate this problem [52,53]. The PVI reduces the influence of background soil by using a soil line [54]. The RVI is suitable for green plants and is highly correlated with the LAI, biomass, and chlorophyll content; however, it requires atmospheric correction before use [55]. The correlation between different vegetation indices and chlorophyll content differs significantly (Figure 5). For example, the correlation between NDVI and GNDVI is high (0.8–1.0), indicating a strong positive correlation with chlorophyll content. RVI is also highly correlated with the chlorophyll content but is sensitive to atmospheric effects. Some indices, such as Cl and CIgreen, have lower correlations (0.5–0.7) with scattered distributions, indicating that environmental conditions significantly affect their sensitivity. PVI and other indices exhibit moderate correlations and are suitable for use in scenarios with significant soil background interference. The advanced index TCARI/OSAVI combines physical models and machine learning through ratio calculations to effectively separate the signals of chlorophyll and leaf area index (LAI) and reduce cross-interference. Generally, NDVI, GNDVI, and RVI provide more accurate estimates of the chlorophyll content, whereas other indices require correction methods and depend on specific conditions to ensure sufficient accuracy and stability [56].

### 3.4. Data Preprocessing

Comprehensive data preprocessing is a critical prerequisite for reliable chlorophyll estimation [57]. It reduces data noise and artifacts, ensuring that the spectral input reflects vegetation signals rather than external interference. Major preprocessing components include:

Noise removal: Random noise from sensors or environmental variability can distort spectral measurements. Median filtering is effective in removing impulsive noise, while wavelet denoising provides multi-resolution smoothing that preserves sharp spectral features [58].

Atmospheric correction: To retrieve accurate surface reflectance, atmospheric effects must be compensated [59]. The 6S radiative transfer model (Second Simulation of the Satellite Signal in the Solar Spectrum) is a widely accepted method for correcting scattering and absorption effects in satellite and airborne remote sensing data [60].

Spectral smoothing: The Savitzky–Golay filter is commonly applied to smooth reflectance curves while preserving critical absorption features [61], thus enhancing the signal-to-noise ratio without distorting spectral information [62]. Together, these preprocessing steps significantly reduce non-biological variability in spectral data and improve the stability, accuracy, and generalizability of downstream chlorophyll.

### 3.5. Extraction and Optimization of Spectral Feature Variables

The extraction and optimization of spectral feature variables play a crucial role in accurately estimating crop chlorophyll content. These methods can be systematically classified into derivative analysis, principal component analysis, and wavelet transform methods.

Derivative-based approaches enhance the sensitivity of spectral reflectance to biochemical properties such as chlorophyll content. The first derivative of reflectance spectra, especially in the red (around 680 nm) and near-infrared (around 750 nm) regions, shows a strong correlation with chlorophyll levels, as it emphasizes the rate of change in reflectance [38]. In contrast, the second derivative is more suitable for detecting spectral inflection points and extracting texture-related information, offering insights into vegetation structure and growth conditions. However, derivative calculations are highly sensitive to noise, necessitating the integration of median filtering and Gaussian filtering to suppress noise and improve signal clarity.

Principal Component Analysis (PCA) is widely employed to reduce redundancy in hyperspectral data and extract meaningful spectral features. It transforms the original data into a new orthogonal coordinate system composed of principal components, which are uncorrelated and ranked by the amount of variance they explain [63]. This technique effectively reduces data dimensionality and computational burden while preserving key spectral information relevant to chlorophyll inversion and vegetation classification.

The wavelet transform method offers a powerful time-frequency analysis framework for remote sensing data. [64]. It enables the decomposition of spectral signals across multiple scales, making it ideal for capturing both low-frequency structural information and high-frequency textural details in remote sensing imagery [65]. Wavelet analysis has demonstrated the ability to enhance inversion accuracy by characterizing subtle variations in vegetation reflectance, especially in complex [66] or heterogeneous environments [67].

## 4. Methods for Chlorophyll Content Estimation

Statistical inversion models, radiative transfer models (RTMs) based on physical methods, and hybrid models incorporating various methods have been used to estimate crop chlorophyll content. The models have different advantages in terms of theoretical basis, applicable scenarios, and inversion accuracy. Choosing the appropriate model is critical to improving inversion accuracy.

### 4.1. Determination of Chlorophyll Content

Detection methods for crop chlorophyll content include chemical reagent extraction and instrument measurements. The former uses solvents, such as acetone or ethanol, to extract chlorophyll. The latter uses a spectrophotometer to measure light absorption at wavelengths of 663.8 nm, 646.8 nm, and 480 nm to calculate the chlorophyll concentration. Chemical reagent extraction requires destructive sampling and is complex [68], making it unsuitable for large-scale estimation [52]. Instruments are relatively easy to operate. For example, the portable SPAD502 instrument determines the relative chlorophyll content by measuring the absorbance difference of plant leaves between two wavelength regions; it is suitable for single-point leaf measurements [69]. The Spectrum CM1000 relative chlorophyll meter [70] determines the relative chlorophyll content of leaves by measuring the transmittance at two wavelengths, 700–840 nm.

### 4.2. Inversion of Crop Chlorophyll Content Based on Measured Data

#### 4.2.1. Statistical Methods

Statistical analysis using measured data is a common method for estimating chlorophyll content (Table 1). In early studies stages, predictions were made by analyzing the linear relationship between spectral data and chlorophyll content. However, as the amount of data increased, the accuracy and stability decreased. Subsequently, stepwise multiple linear regression (SMLR) [71], principal component regression (PCR) [72], and partial least squares regression (PLSR) were utilized [16]. These methods are more suitable for removing redundant information and extracting the most important variables, significantly improving model accuracy and stability. They became mainstream methods in the 1980s [11]. As data complexity increased, ridge regression (RR) and the least absolute shrinkage and selection operator (LASSO) were developed to minimize multicollinearity. These methods utilize regularization terms to reduce model complexity, avoid overfitting, and improve prediction stability and accuracy [73]. However, these methods typically require a large amount of measured data, and their prediction accuracy may decrease when applied to other crops or different environments. Therefore, maintaining high-accuracy predictions across different scenarios remains a challenge.

#### 4.2.2. Machine Learning Algorithms

Machine learning methods have high prediction accuracy and stability due to efficient variable selection and optimization. They are suitable for different crop types and study areas and have high estimation efficiency (Table 2).

In the early stages of machine learning development, artificial neural networks (ANNs) were widely used in remote sensing estimation of vegetation chlorophyll content due to their ability to handle complex nonlinear problems [76] and model local data structures [77]. As the remote sensing data volume increased, deep neural networks (DNNs) could better capture the complex nonlinear relationships between remote sensing data and chlorophyll content by learning from large-scale datasets, improving inversion accuracy [78]. Ensemble learning (EL) methods combine multiple learners, improving the model’s ability to predict chlorophyll content. For example, bagging [79] and random forest regression (RFR) reduce the prediction variance by training multiple models and averaging their predictions [80]. Boosting methods further reduce bias by generating multiple weak learners and combining them with weights. The most widely used method is gradient boosting regression trees (GBRTs), which has achieved good results in remote sensing inversion of crop chlorophyll content [81].

Kernel machine learning methods, such as support vector regression (SVR) [82,83] and Gaussian process regression (GPR), use kernels to map input data onto high-dimensional spaces, enabling the effective modeling of complex nonlinear relationships in higher-dimensional spaces [84]. GPR estimates the mean and variance of chlorophyll content prediction using Bayesian methods, enabling uncertainty quantification [85].

However, machine learning methods for crop chlorophyll inversion have several disadvantages. Their performance depends largely on the quantity and quality of training data. Models using insufficient or inaccurate training data may exhibit overfitting or underfitting, reducing prediction accuracy. Furthermore, unlike statistical and physical models, machine learning models cannot be used to explain the underlying mechanisms of chlorophyll content changes and determine the mapping relationships between crop physical properties and spectral data.

#### 4.2.3. Deep Learning Methods for Chlorophyll Retrieval

Deep learning methods, particularly convolutional neural networks (CNNs), recurrent neural networks (RNNs), and Transformer-based models, have shown significant promise in chlorophyll content estimation from remote sensing data due to their ability to model complex nonlinear and high-dimensional relationships [78].

One-dimensional CNNs (1D-CNNs) effectively extract spectral features from hyperspectral data, while two-dimensional CNNs (2D-CNNs) capture spatial patterns in UAV or satellite imagery [86,87,88]. For instance, 1D-CNN achieved R^2^ = 0.94 for leaf chlorophyll content, outperforming traditional machine learning approaches [86]. CNNs have also been combined with radiative transfer model simulations to improve robustness [89].

CNN-LSTM hybrid models leverage both spatial–spectral features and temporal sequences to track chlorophyll dynamics throughout the growing season. Zhang et al. (2024) developed a CNN-BiLSTM model, achieving R^2^ = 0.81 in winter wheat yield estimation by fusing spectral indices and solar-induced fluorescence [90]. Similarly, LSTM models trained on multi-temporal satellite data have shown superior performance in aquatic chlorophyll-a prediction, with RMSE = 0.63 mg/m³ and R^2^ = 0.98 [91].

Transformer architectures offer advantages in handling long-range dependencies. Sun et al. (2024) proposed a Transformer-based model, ChloroFormer, for chlorophyll-a forecasting, outperforming other deep learning models, particularly during algal bloom periods. Their attention-based architecture effectively modeled complex periodic signals [92]. CNN–Transformer fusion networks have also been explored for hyperspectral land-cover tasks, showing potential for chlorophyll applications [93].

Despite strong performance (R^2^ > 0.8; RMSE lower than traditional models), deep learning models face limitations including large data requirements, generalization issues, and lack of interpretability [94]. Physics-informed strategies are emerging to address these limitations, using radiative transfer models like PROSAIL to generate synthetic training data [89]. Additionally, explainable AI methods such as Grad-CAM and SHAP help improve model transparency [90].

Overall, deep learning offers great potential for chlorophyll retrieval. However, further research is needed to address challenges such as data requirements, model interpretability, and generalization capabilities [94].

### 4.3. Inversion of Crop Chlorophyll Content Based on Radiative Transfer Modeling

RTMs simulate light scattering, absorption, and emission through the atmosphere. They consider multiple physical processes, such as solar and surface radiation, to model the spectral radiative characteristics of surface objects [95]. They are used to derive surface reflectance and transmittance by modeling radiative processes in the vegetation canopy, enabling the estimation of the chlorophyll concentration. They are used for the quantitative inversion of remote sensing data [96].

In the early development of RTMs, the SAIL model [97] was used to determine the bidirectional canopy reflectance. It is based on the Suits model [98] and has become a classic plant canopy reflectance model. The subsequent SAILH model considered the hotspot effect and multiple scattering [99], making it more representative of the canopy structure. Leaf-level models, such as the PROSPECT model [100], use a layered approach to simulate the optical properties of leaves and are suitable for wavelengths ranging from 400 to 2500 nm. PROSAIL integrates PROSPECT and SAIL and is a widely used RTM [101]. It has become the core tool in more than 70% of crop trait studies. The PROSAIL model, sample databases, and iterative optimization algorithms have been used to invert the chlorophyll content of crops, such as potatoes [102,103].

Traditional RTMs are often simplified into two-dimensional models. However, three-dimensional RTMs have gained more attention in recent years because they accurately describe spatial heterogeneity. Three-dimensional models, such as RGM (Radiative Growth Model), RAPID (Radiative transfer model with Accelerated Processing and Innovative Dynamics), Raytran (Ray Tracing Radiative Transfer Model), and DART (Discrete Anisotropic Radiative Transfer), can simulate high-resolution images and represent complex scenarios [104]. For example, the DART model has been used to assess the contributions of various components of the tree canopy’s spectral response to the results [32]. In 2021, a novel two-step inversion model to invert leaf reflectance using LAI estimated from Sentinel-2 MSI data and to estimate chlorophyll content at forest observation sites [105].

### 4.4. Mixed Methods for Inverse Modeling of Crop Chlorophyll Content

The integration of radiative transfer models (RTMs) and machine learning (ML) has emerged as a robust framework for chlorophyll content inversion, addressing the limitations of standalone physical or statistical approaches. For example, the PROSAIL-RF hybrid model [106] combines PROSAIL-simulated spectral libraries with random forest regression to estimate chlorophyll content in apple orchards, achieving an R^2^ of 0.85 and reducing computational time by 40% compared to traditional RTM inversion. Similarly, a two-step hybrid approach [107] first derives leaf optical properties from Sentinel-2 data using PROSAIL, then applies gradient boosting to map chlorophyll at regional scales (R^2^ = 0.88, RMSE = 8.2 µg/cm^2^). Recent advancements include physics-informed neural networks [89], where PROSAIL-generated synthetic data trains a 1D-CNN, enabling accurate retrieval under varying canopy structures (R^2^ > 0.9). Key advantages of hybrid methods include:

1. Enhanced interpretability: Physical constraints from RTMs guide ML training, avoiding “black-box” pitfalls.
2. Scalability: GPU-accelerated RTMs (e.g., DART) enable large-area applications.
3. Adaptability: Fusion with UAV hyperspectral data improves resolution to 5 cm for precision farming.

## 5. Evaluation of Modeling Results

A systematic analysis of 67 studies from the 788 retrieved articles revealed critical insights into the performance of remote sensing inversion models for crop chlorophyll content. The accuracy of these models was evaluated based on three dimensions: modeling methods, data sources, and crop specificity.

### 5.1. Model Performance Across Methodologies

**Figure 6:**
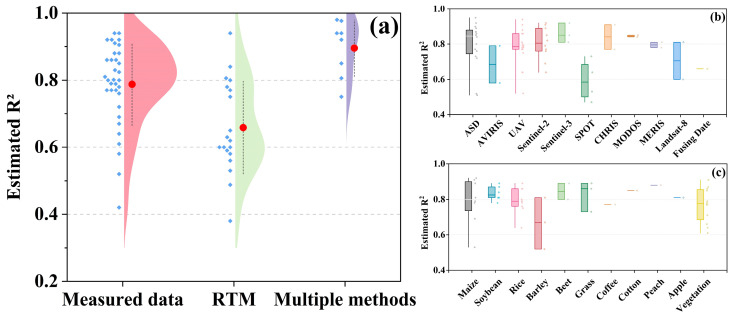
Comparison of chlorophyll inversion accuracy. (**a**) Different modeling methods; (**b**) different data sources; (**c**) different crops.

As shown in Figure 6a, models relying on ground-truth data achieved the highest accuracy (average R^2^ = 0.90, range: 0.85–0.95), attributed to their direct calibration with field measurements. For example, machine learning models (e.g., random forest and SVR) demonstrated exceptional performance in homogeneous croplands, with R^2^ values exceeding 0.9 for staple crops like maize and rice [4,69]. However, their accuracy declined in heterogeneous environments (e.g., mixed cropping systems) due to spectral interference from non-target vegetation [28].

Radiative transfer models (RTMs), while theoretically robust (average R^2^ = 0.75) showed lower accuracy in dense canopies or under cloudy conditions. For instance, PROSAIL exhibited R^2^ reductions of 15–20% in orchards with complex 3D structures, as leaf angle distribution and shadow effects were inadequately parameterized [7]. Hybrid models combining RTMs with machine learning (average R^2^ = 0.85) mitigated these limitations by integrating physical constraints with data-driven adaptability. A notable example is the two-step inversion framework proposed [105], which first derived leaf reflectance from Sentinel-2 data and then estimated chlorophyll content using a gradient-boosting algorithm, achieving R^2^ > 0.88 across diverse crops.

### 5.2. Impact of Data Sources on Accuracy

The 67 papers analyzed in this study all used public datasets (Figure 6b). Sentinel-2 has a red edge band (705 nm, 740 nm) and high spatial resolution (10 m), and its average accuracy (R^2^ = 0.85) is significantly better than Landsat-8 (R^2^ = 0.78) and Gaofen series satellites (R^2^ = 0.72). Its red-edge bands (705 nm and 740 nm) were particularly effective in capturing chlorophyll dynamics in wheat and soybean [40]. Drone-based data, despite ultra-high resolution (cm-level), showed variable accuracy (R^2^ = 0.75–0.85) depending on flight altitude and sensor calibration. For instance, MicaSense RedEdge achieved R^2^ = 0.82 in rice paddies but dropped to 0.68 in maize fields with tall canopies due to shadow interference [21]. MODIS data, though suitable for global monitoring (R^2^ = 0.65–0.75), faced challenges in smallholder farms where mixed pixels diluted spectral signals [42].

### 5.3. Crop-Specific Variations and Environmental Dependencies

Crop type significantly influenced model generalizability (Figure 6c). Models for monocot crops (e.g., rice, wheat) outperformed those for dicots (e.g., soybean, tomato), with R^2^ differences of 0.1–0.2. This disparity stems from structural variations: monocots’ uniform canopy geometry simplified reflectance modeling, while dicots’ irregular leaf arrangements introduced spectral noise [32]. Environmental factors further modulated accuracy. For example, in arid regions, soil background reflectance (e.g., high sand content) reduced NDVI-based model accuracy by 10–15%, necessitating soil-adjusted indices like SAVI [55]. Conversely, in humid tropics, cloud cover, and atmospheric water vapor attenuated satellite signals, requiring fusion with ground or drone data for correction [46].

### 5.4. Limitations and Optimization Pathways

Current models face three key limitations: (1) Overfitting in machine learning: Models trained on localized datasets often failed in cross-regional applications. For instance, a random forest model calibrated in temperate China (R^2^ = 0.92) dropped to R^2^ = 0.68 when applied to subtropical India, highlighting the need for geographically diverse training data [108]. (2) Computational inefficiency in RTMs: PROSAIL inversions required 10–15 min per hectare, limiting real-time applications [31]. Recent advances in GPU-accelerated RTMs reduced processing time by 80%, enabling near-real-time monitoring [107]. (3) Sensor interoperability: Disparities in spectral band definitions across platforms (e.g., Sentinel-2 vs. Landsat) complicated multi-source fusion. Standardized band alignment protocols, as proposed by Zhu et al. (2018), could mitigate this issue [44].

### 5.5. Cost-Benefit Analysis of Data Sources

While drone data provided unparalleled spatial detail, its cost (approximately $500/km^2^) was 10-fold higher than satellite data [38]. Hybrid approaches, such as combining Sentinel-2 with periodic drone flights, balanced cost, and accuracy. For example, a biweekly drone–Sentinel fusion strategy achieved R^2^ = 0.87 at 60% lower cost than pure drone-based monitoring [20].

## 6. Conclusions and Outlook

Remote sensing-based chlorophyll estimation in crops has advanced remarkably in recent years (2018–2024), driven by innovations in sensors, algorithms, and interdisciplinary collaboration. Modern multispectral and hyperspectral instruments—from satellite platforms like Sentinel-2 to UAV-based imagers—now provide spectral bands and resolutions finely tuned to vegetation signals. In particular, the red-edge region around 705–740 nm has proven highly sensitive to foliar chlorophyll variations, enabling empirical models to achieve very high accuracy (often R^2^ > 0.85) in estimating leaf chlorophyll content under controlled conditions.

Building on this foundation, the field has evolved toward more advanced modeling approaches. Hybrid methods that combine radiative transfer models (RTMs) such as PROSAIL with machine learning algorithms have emerged as powerful tools, significantly enhancing both accuracy and generalizability across different crops and environments. These models often achieve R^2^ values exceeding 0.90, especially when red-edge and derivative spectral features are used. Physics-guided machine learning further enhances interpretability and robustness, marking a paradigm shift from purely empirical regressions toward biophysically meaningful retrievals.

However, significant challenges remain. Model performance often degrades when applied to unseen crop types or new environments, particularly due to differences in leaf structure, soil background, or viewing geometry. Sensor discrepancies and inconsistent preprocessing protocols hinder multi-source data integration, while RTM-based simulations can be computationally demanding. Moreover, deep learning methods require large, diverse labeled datasets, the lack of which limits operational scalability.

Looking ahead, the integration of hyperspectral, thermal, LiDAR, and fluorescence data offers a promising pathway for capturing the multi-dimensional characteristics of crop health. Lightweight models deployed via UAVs or IoT sensors, combined with edge computing, could enable real-time chlorophyll monitoring. Simultaneously, the establishment of standardized spectral libraries, global benchmarking datasets, and transfer learning frameworks will be vital for improving model scalability and generalization.

In conclusion, remote sensing has demonstrated great potential for large-scale, accurate, and timely estimation of chlorophyll content in crops. Continued progress depends on interdisciplinary integration—linking agronomic insights, remote sensing technologies, and AI-driven modeling. Future work should prioritize the development of scalable, interpretable, and globally applicable frameworks that bridge the gap between research and operational agricultural monitoring.

## Figures and Tables

**Table 1:** Advantages and disadvantages of linear nonparametric methods.

Methods	Advantages	Disadvantages
SMLR (stepwise multiple linear regression)	(1) Simple, fast, and easy to use. (2) Screens a large number of potential predictors to obtain the best one.	(1) Multicollinearity occurs in canopy hyperspectral data. (2) The selected wavelength is often not related to the absorption characteristics of the targets [74,75].
PCR (principal component regression)	(1) Mitigates multicollinearity and avoids overfitting. (2) Improves the predictive performance and provides stable regression coefficients.	(1) Does not consider the response variable when eliminating principal components and relies only on the magnitude of the variance of components. (2) Does not perform feature selection. (3) Low interpretability.
PLSR (partial least squares regression)	(1) Handles multiple inputs and outputs, data noise, and missing data.	(1) Considers the cross-product relations with the response variables and is not based on the (co)variances between independent variables. (2) Difficult to interpret. (3) Response distribution is unknown.
RR (ridge regression)	(1) Reduces overfitting. (2) Adds bias to estimators to reduce the standard error. (3) Uses all predictors in the final model.	(1) Low model interpretability. (2) Does not perform feature selection. (3) Uses variance instead of bias.
LASSO	(1) Reduces overfitting. (2) Fast in terms of inference and fitting.	(1) Arbitrary variable selection. (2) Difficulty in selecting predictors. (3) Uses a small bias since the prediction depends on the variable. (4) Lower prediction performance than RR.

**Table 2:** Advantages and disadvantages of machine learning methods.

Methods	Advantages	Disadvantages
ANNs	(1) Suitable for determining correlations between variables and data distribution. (2) Not affected by noise. (3) DNNs have high computational power DNN. (4) Reduces overfitting.	(1) Difficult to interpret the results and performance of this black-box model. (2) Requires high computational power. (3) Needs many data for training. (4) Difficult to optimize the neural network model.
EL	(1) Reduces variance and bias. (2) Uses weak learners. (3) Insensitive to data distribution and noise.	(1) Difficult to interpret the results. (2) Predictive accuracy may be reduced by choosing an incorrect model.
SVR	(1) Robust to overfitting. (2) Handles nonlinear and high-dimensional data. (3) A slight change in the data does not affect stability or the hyperplane.	(1) Does not compute the uncertainty of prediction. (2) High computational complexity. (3) Not suitable for large datasets and sensitive to noise. (4) Choosing the optimal kernel is critical.
GPR	(1) Captures model uncertainty by calculating the mean and standard deviation of prediction. (2) Does not require a large sample size for training, and the sample size is unrelated to the data distribution. (3) Incorporates expert knowledge on the model type by using a kernel.	(1) Computationally expensive when using a large sample size. (2) The efficiency is relatively low in high-dimensional spaces.
KRR	(1) Faster computation than SVR and GPR. (2) Straightforward model training because it determines the parameters that reduce the mean squared error.	(1) No sparseness in the vector of coefficients, unlike the SVR.

## Data Availability

All cited references are listed in Web of Science.
